# Cardiovascular effects of β-blockade in a sheep model of severe sepsis

**DOI:** 10.1186/cc10405

**Published:** 2011-10-27

**Authors:** P Calzavacca, R Ramchandra, L Booth, R Bellomo, CN May

**Affiliations:** 1 Howard Florey Institute, University of Melbourne, Parkville, VIC, Australia; 2 Department of Anaesthesia and Intensive Care, AO Melegnano, PO Uboldo, Cernusco sul Naviglio, Italy; 3 Department of Intensive Care and Department of Medicine, Austin Health, Melbourne, VIC, Australia

## Introduction

In sepsis, sympathetic nerve activity is differentially increased in individual organs. The increased cardiac sympathetic nerve activity is partly responsible for the increase in heart rate (HR) and cardiac output (CO) opposing the development of hypotension [1]. Recently, in a rat septic model, β-blockade appeared safe and decreased the inflammatory response and mortality [2]. Accordingly, we sought to investigate the cardiovascular effects of selective β1-receptor blockade in a sheep model of sepsis.

## Methods

Eight merino ewes were studied in a university-affiliated research institute in Melbourne. The study design was a prospective interventional crossover animal study. The animals had renal and cardiac flow probes implanted to continuously measure CO and renal blood flow (RBF). Every animal was randomly allocated to receive sepsis and atenolol (atenolol group, AG) or sepsis alone (control group, CG) and then crossed over. After 24 hours of baseline period, sepsis was induced through a bolus of live *Escherichia coli *by a continuous infusion for a total 24 hours of sepsis. After the first 8 hours of sepsis (development sepsis period, DS), a bolus of atenolol (10 mg bolus) was given followed by a continuous infusion of 0.125 mg/kg/hours for 16 hours. Two-way repeated-measure ANOVA was performed to compare the average of periods and group interaction. *P *< 0.05 was considered significant (not significant (NS), *P *> 0.05).

## Results

**Figure 1:**
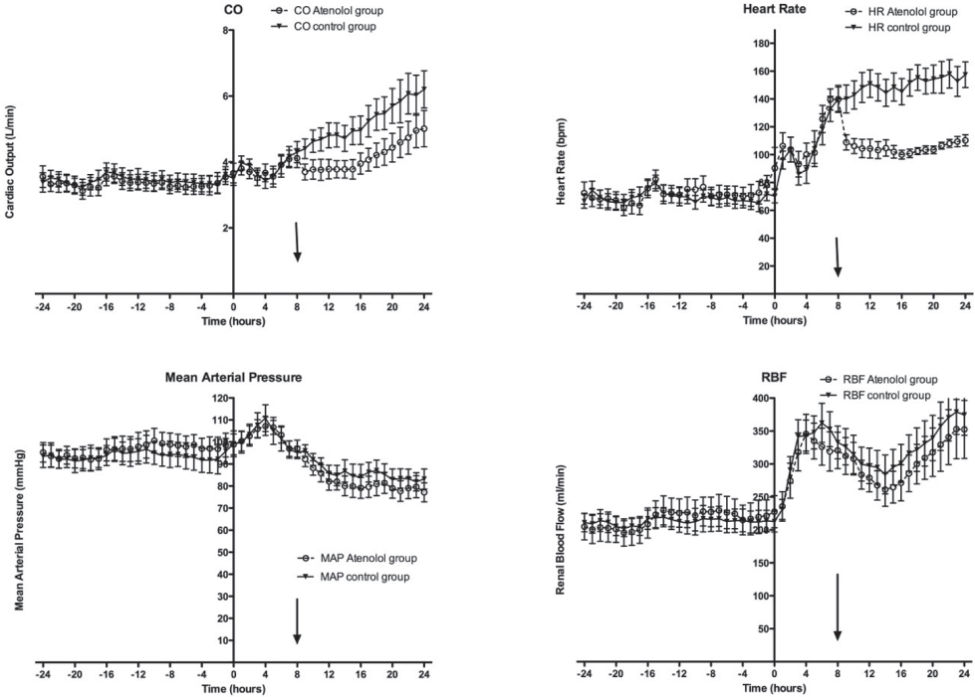
**Hemodynamic variables during baseline and sepsis in the atenolol (AG, white circles, dotted line) and control (CG, black triangles, continuous line) groups**.

Animals in the AG and CG had similar baseline values and developed a similar hyperdynamic state in the DS (Figure 1 and Table 1). Atenolol reduced CO and HR without changes in stroke volume. Hypotension was slightly greater in the AG than in the CG (MAP: 81.5 vs. 86.1 mmHg) with a greater decrease in total peripheral conductance (16.8 vs. 22.1 l/minute/mmHg). Changes in lactate level were similar. Similar increases in RBF and in renal vascular conductance (RVC) were observed in the AG and CG and after an initial increase in diuresis in the DS, oliguria similarly subsequently developed in both groups. Creatinine clearance decreased in a similar way in the AG and CG from 59.2 (± 2.8) to 32 (± 5.7) ml/minute and from 65.2 (± 9.9) to 36 (± 7) ml/minute, respectively (*P *= 0.381). One animal in the AG and two in the CG died in the 24 hours after the end of sepsis.

**Table 1:** Hemodynamic and renal findings during baseline, development (DS) and intervention sepsis periods in the CG and AG groups

	Group	Baseline period	Development sepsis period (DS)	Sepsis intervention period	*P *value
CO	CG	3.68 (0.29)	3.68 (0.29)	4.12 (0.29)	**<0.001**
	AG	3.21 (0.22)	3.21 (0.22)	5.63 (0.53)	
HR	CG	66.0 (6.5)	66.0 (6.5)	108.8 (8.6)	**<0.001**
	AG	59.7 (6.4)	59.7 (6.4)	111.6 (8.4)	
MAP	CG	93.7 (5.1)	102.5 (3.6)	86.1 (4.1)	**0.035**
	AG	96.3 (5.0)	102.5 (3.8)	81.5 (4.3)	
RBF	CG	217.3 (14.8)	217.3 (14.8)	324.8 (19.7)	0.194
	AG	214.7 (19.9)	214.7 (19.9)	292.3 (27.4)	
UO	CG	31.1 (7.1)	84.1 (20.4)	22.1 (4.8)	0.097
	AG	34.8 (7.0)	52.3 (17.4)	16.8 (11.0)	
TPC	CG	38.2 (3.4)	38.9 (3.4)	63.9 (6.8)	0.084
	AG	34.3 (3.1)	37.3 (3.2)	51.4 (6.8)	<0.001
SV	CG	51.4 (3.7)	39.1 (3.6)	35.5 (8.2)	0.147
	AG	51.2 (4.6)	36.7 (3.5)	39.6 (3.6)	
RVC	CG	2.39 (0.18)	3.26 (0.26)	3.74 (0.41)	0.55
	AG	2.23 (0.16)	2.88 (0.26)	3.53 (0.44)	

Values are the mean (± standard error). *P *value: two-way repeated-measures ANOVA interaction between treatment group and time (see text for definitions).

## Conclusion

β-blockade in hyperdynamic sepsis appears safe. It results in only limited decreases in mean arterial pressure, and does not increase lactate levels or worsen renal function.
